# Influence of *β*-Ionone in the Phytotoxicity of the Rhizome of *Iris pallida* Lam

**DOI:** 10.3390/plants13020326

**Published:** 2024-01-22

**Authors:** Yourk Sothearith, Kwame Sarpong Appiah, Chhin Sophea, Jady Smith, Say Samal, Takashi Motobayashi, Yoshiharu Fujii

**Affiliations:** 1 Department of International Environmental and Agricultural Science, Tokyo University of Agriculture and Technology, Saiwai-cho 3-5-8, Fuchu 183-8509, Tokyo, Japan; ksappiah90@gmail.com; 2 Ministry of Environment, Morodok Techcho (Lot 503) Tonle Bassac, Chamkarmorn, Phnom Penh 120101, Cambodia; sopheachhin@gmail.com (C.S.); officeofssa@gmail.com (S.S.); 3 Department of Crop Science, University of Ghana, Legon, Accra P.O. Box LG 44, Ghana; 4 Centre for Biodiversity Conservation, Royal University of Phnom Penh, Russian Federation Boulevard, Toul Kork, Phnom Penh 120404, Cambodia; 5 Forest Research Institute, University of the Sunshine Coast, Sippy Downs, QLD 4556, Australia; 6 Ministry of Land Management, Urban and Construction, Lot 2005, Street 307, Sangkat Khmuonh, Khan Sen Sok, Phnom Penh 120803, Cambodia

**Keywords:** allelopathy, allelochemicals, total activity, specific activity, inhibitory, phytotoxicity

## Abstract

*Iris pallida* Lam., also known as Sweetie Iris, is a perennial ornamental and medicinal plant that produces a wide range of secondary metabolites. The Sweetie Iris was recently reported to have high allelopathic properties with the potential to be explored in sustainable weed management. This study aimed to identify and evaluate the contributions of compounds involved in the inhibitory effects of the rhizome of Sweetie Iris. High-performance liquid chromatography (HPLC) analysis was used to determine the content of *β*-ionone in the rhizome of Sweetie Iris. The phytotoxicity of *β*-ionone was evaluated on lettuce (*Lactuca sativa* L.) and other test plants. The content of *β*-ionone in the crude extract of Sweetie Iris rhizome was found to be 20.0 mg g^−1^ by HPLC analysis. The phytotoxicity bioassay showed that *β*-ionone had strong inhibitory activity on the growth of lettuce (*Lactuca sativa* L.) and the other test plants, including *Taraxacum officinale*, *Stellaria media*, *Eleusine indica*, *Amaranthus hybridus*, *Vicia villosa,* and *Brassica napus*. At a concentration of 23.0 µg mL^−1^, *β*-ionone inhibited the growth of all test plant species treated. Therefore, *β*-ionone is an active compound among the other allelopathic substances contained in the rhizome of Sweetie Iris.

## 1. Introduction

Higher organisms including plants, algae, bacteria, and fungi produce certain bioactive secondary metabolites which can influence (including positive or negative effects) the growth and development of other organisms in a natural ecosystem in a phenomenon called allelopathy [1]. The bioactive secondary metabolites that interfere in the growth and development of other plants are called allelochemicals [2]. Allelochemicals are mostly released from plant tissues through volatilisation or leaching from aerial parts, exudation from roots, and decomposition of plant residues in soil, while there are different kinds of bioactivity and modes of action, the related compounds commonly share similar biosynthetic pathways; however, some metabolites can be produced using diverse biosynthetic pathways [3,4]. Several plants have been reported with plant growth inhibitory potentials, but only a few have shown strong allelopathic effects [5,6]. Current research has focussed on the search for novel compounds from natural plants with demonstrable herbicidal activity to promote sustainable agriculture [7,8], particularly to respond to the increasing demand for organic products over the last decade [9]. The pharmacological properties of medicinal plants were reported to contain biological functions and a strong relationship to allelopathic activity, particularly in ecological weed management [10,11,12,13]. Additionally, the bioactive constituents from medicinal plants produce some physiological effects on humans, animals, and plants in natural ecology [14,15]; some allelochemicals responsible for the growth activities were isolated and identified, such as artemisinin from *Artemisia annua* [16], ethyl 2-methylacetoacetate from *Phragmites communis* [17], safranal from *Crocus sativus* [18], L-3,4-dihydroxyphenylalanine (L-DOPA) from *Mucuna pruriens* [19], and rutin from *Fagopyrum esculentum* [20], carnosic from *Rosmarinus officinalis* [21], and cyanamide in *Vicia villosa* [22]. These allelopathic substances can play a crucial role as natural herbicides and can help resolve problems like pest biotypes, health defects, soil, and environmental pollution resulting from the indiscriminate use of synthetic agrochemicals [23]. Weeds pose a more serious threat to crops than other pest species, with up to 50% losses of productive crops expected in Asia, and other continents, if weeds are not properly controlled [24,25,26]. The use of allelopathic species as a cover crop can mitigate weed infestation, insect pests, and disease pathogens as well as provide more fertility and organic matter to enhance soil quality and farm yields [27], with traditional breeding or genetic engineering methods enhancing the biosynthesis and release of allelochemicals [28].

Irises are a group of plants with immense medicinal value, used in the treatment of cancer, inflammation, and bacterial and viral infections [29]. The phytochemicals such as flavones, flavone C-glycosides, isoflavones, terpenoids, xanthones, phenolics, stilbenes, and quinones have been reported among this group of plants [30,31,32]. Although the irises were found to be important sources of isoflavones, *Iris pallida* Lam. has gained more attention among other species for recent products such as perfume and essential oil [33,34]. *Iris pallida*, also known as Sweetie Iris, belongs to the Iridaceae family and is native to Croatia. The Sweetie Iris is a perennial herb and is mostly cultivated for essential oil, aromatherapy, and traditional medicine [30,35]. Several parts of *Iris pallida* including rhizome, leaves, and flowers produce a wide range of secondary metabolites and some phenolic and volatile compounds were also reported, such as squalene from the leaves; isoflavones from the rhizome; and terpenes, alcohols, and esters from the flowers [36,37,38]. Triterpenoids from iris rhizomes have been shown to be the precursors of irones. The aromatic principles from iron extracts have been used in many industries, and the most precious constituents also respond in characteristic scent [29]. *Iris pallida* was recently reported to have a strong plant growth inhibitory effect exhibited through both leaches and volatiles. By using the sandwich method, the amount of 10 mg of *Iris pallida* showed high inhibitory effects on the radicle and hypocotyl elongation percentages (4% and 7.1%, respectively). Additionally, *Iris pallida* also showed an inhibitory effect on the growth of lettuce in the range of 22.1% and 6.7% for radicle and hypocotyl elongation, respectively, by using the dish park method [39,40]. However, its allelopathic substances were yet to be reported. This study, therefore, aimed to identify the allelochemical presented in the Sweetie Iris, including the contributions of its detected compounds to the phytotoxicity of plant growths.

## 2. Results and Discussions

### 2.1. The Content of β-Ionone in Sweetie Iris

**Figure 1:**
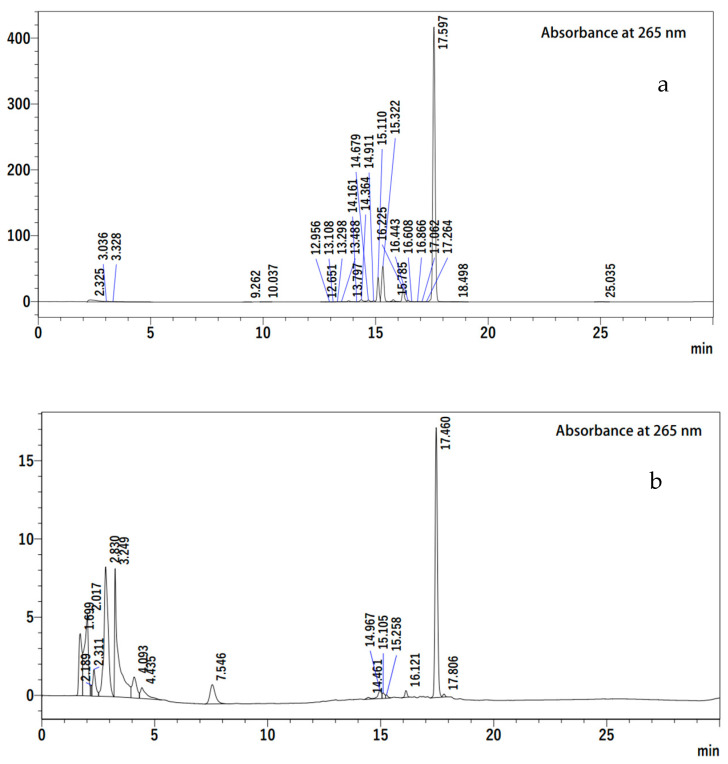
High-performance liquid chromatography analysis of (**a**) *Iris pallida* Lam. and (**b**) *β*-ionone.

The identification and quantification of *β*-ionone in the crude extract of the Sweetie Iris were conducted using uigh-performance liquid chromatography (HPLC) analysis. The content of *β*-ionone was detected in the high peak areas during 17 min (Figure 1). The concentration of *β*-ionone in Sweetie Iris crude extract was found to be 20.0 mg g^−1^ based on the calibration curve. Beta-ionone (4-(2,6,6-trimethylcyclohex-1-en-1-yl) but-3-en-2-one) is a type of terpene compound produced by the degradation of carotenoids [41]. This prominent scented and aromatic molecule is present in several plant species such as the leaves of *Iris pallida* (Iridaceae) [33], the flowers of *Osmanthus fragrans* (Oleaceae) [42], and other Rosaceae families such as *Rosa bourboniana* and *Rosa canina* and other fruits [43]. Additionally, *β*-ionone is also found at a different concentration from the essential oil of the leaves of *Lawsonia inermis* (Lythraceae) [44], the flower of *Rosa moschata* (Rosaceae) [45], the aerial parts of *Viola tricolor* (Violaceae) [46], the flower of *Medicago marina (Fabaceae)* [47], and the maca root of *Lepidium meyenii* (Brassicaceae) [48].

**Figure 2:**
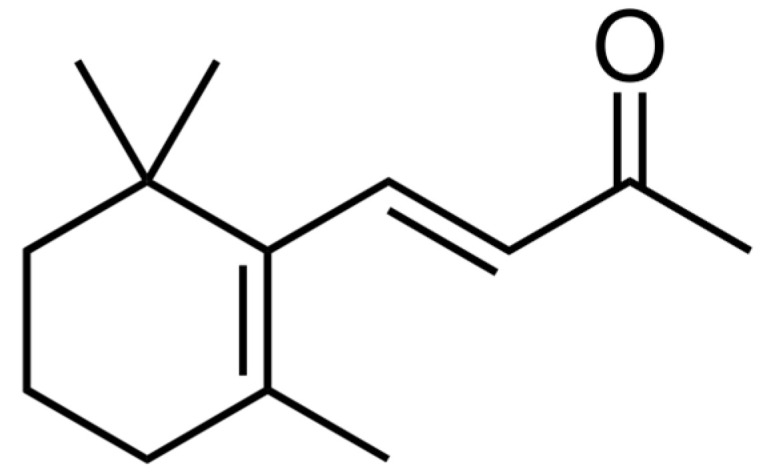
Chemical structure of *β*-ionone.

The concentration of *β*-ionone was highest in corn, tea, and carrots and found in smaller concentrations in hyssops, peppermints, and safflowers [49]. The content of *β*-ionone as in Figure 2 was synthesised in early 1893 to clarify the structure of irones, a key flavor compound of *Iris pallida* [50]. *β*-ionone is an intermediate key in the synthesis of vitamins A, E, and K [51,52]. It has significant physiological and biological activities such as antioxidant, antimutagenic, and antifungal were also reported [53,54]. *β*-Ionone is a defence compound among other plant apocarotenoids. It serves as an ecological cue, insect attractant, or repellent, and possesses antibacterial and fungicidal properties [55,56,57]. Other studies have also revealed that the content of *β*-ionone has antimicrobial effects on some pathogenic plants [58,59,60] and possesses inhibitory activity against *Microcystis aeruginosa* at high concentrations [61].

### 2.2. Inhibitory Effects of the Crude Extract and Estimated Contribution of β-Ionone in the Phytotoxicity of Sweetie Iris

**Figure 3:**
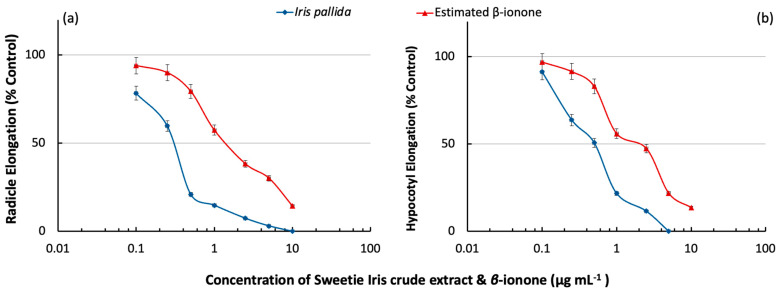
Estimated contribution of *β*-ionone in the Sweetie Iris crude extract on (**a**) the radicle and (**b**) hypocotyl of lettuce growth. Each datum was an average of three replications.

In allelopathy, the contribution of compounds acting as allelochemicals is based on their concentration and inhibition activity (specific activity). The specific activity or EC_50_ is defined as the effective concentration of a compound that causes half-maximal inhibition. Hence, the compounds with high specific activity could be potentially exploited as natural herbicides [62,63]. On the other hand, the total activity of a compound is a function of its specific activity and concentration in the plant. Through this value, the influence of allelopathic effects can be estimated [64]. Therefore, the inhibitory effect of Sweetie Iris in the bioassay was tested on lettuce growth (Figure 3).

The specific activity (EC_50_) of the crude extract was determined to be 0.3 and 0.4 mg mL^−1^ for lettuce radicle and hypocotyl, respectively. The application of 10 mg mL^−1^ of the Sweetie Iris crude extract caused a maximum inhibition of lettuce radicle growth of 96%. However, there is no adverse effect on seed germination at this concentration. The inhibitory effect of *β*-ionone was also tested on the growth of lettuce to compare and estimate its significant contributions to the inhibitory effect of Sweetie Iris crude extract (i.e., total activity). The total activity estimation approach of plant growth inhibition based on concentration and inhibitory effect (specific activity or EC_50_) has been adopted to identify many important allelochemicals such as rutin, cyanamide, juglone, angelicin, L-DOPA, and durantanins [20,21,63,65,66,67]. Total activity estimation based on Sweetie Iris was calculated. For instance, the content of *β*-ionone in 1.0 mg mL^−1^ of Sweetie Iris was determined to be 20.0 µg mL^−1^. The lettuce radicle elongation percentage as a result of the 20.0 µg mL^−1^ of *β*-ionone (estimated amount in the Sweetie Iris) was determined to be 20%. Hence, the equivalent elongation inhibitory effect of the estimated *β*-ionone in the 1.0 mg mL^−1^ of the Sweetie Iris extract was calculated to be 60%. Following the same calculation approach, the inhibitory effect of the Sweetie Iris crude extract on the growth of lettuce was explained by the contribution of *β*-ionone (Figure 3). Hence, *β*-ionone was estimated to be a candidate among other responsible compounds contained in the Sweetie Iris crude extract.

### 2.3. Inhibitory Effects of β-Ionone on the Radicle and Hypocotyl Elongation of Other Plant Species

The growth inhibition on radicle and hypocotyl elongation of other test plants treated with *β*-ionone was also evaluated. The plant species were selected because they are reported to have a significant impact on the environment and agricultural production. Among the selected species include *Taraxacum officinale* L., also known as Dandelion, which is a well-known herbaceous perennial plant in the Asteraceae family. This species is native to Europe and is considered an aggressive invasive species worldwide [68]. Dandelion is drought resistant, adapts to a wide range of light and shade intensity, and can grow in a wide range of soil types and pH levels [69]. Moreover, its ability to colonise a wide range of habitats increases due to the presence of phenotypic plasticity [70]. *Stellaria media* (L.) Villars better known as a Chickweed from the Caryophyllaceae family is a Polycarpic winter annual distributed to compete planted crops in cultivation fields [10,71,72]. Without interference from other plants, the Chickweed can produce around 800 seeds and it takes 7 to 8 years for the seed bank (supply of viable seeds in soil) to be 95% depleted, insuring an infestation for many years [73]. Chickweed contains heterogeneous populations represented by different age classes under natural conditions. *Eleusine indica* (L.), which belongs to the Poaceae family, was listed as one of the five most noxious weeds worldwide. *Eleusine indica* known as Goosegrass or Wiregrass impacts 46 crops in more than 60 countries. A single plant of Goosegrass can produce about 140,000 seeds and spread out rapidly. It was reported that a density of 11.6–19.2 Goosegrass plant m^−1^ of the row reduced 50% of cotton yield loss according to the hyperbolic decay regression model [74,75]. The leaf of Goosegrass was also reported to contain volatile allelopathic compounds [40].

Another selected species was *Amaranthus hybridus* L., commonly called Amaranth or Pigweed, which is a weedy species that belongs to the Amaranthaceae family and is native to Eastern-North, Central, and Northern-South America. The leaves of green Amaranth can grow up to 20 cm long and the entire plant can grow easily up to 3 m in height. A single gram of *Amaranthus hybridus* may contain 3000 seeds and also can produce from 100,000 to 600,000 seeds if given space and time [76,77]. The weedy biotype of *Amaranthus hybridus* has been found to reduce corn and soybean yields in southern Ontario, Canada [78]. The Pigweed can quickly formulate a canopy over shorter vegetables and compete with taller crops, just 1 to 3 green Amaranth plants in 3 m of a corn or soybean row could cause significant yield loss [79]. *Vicia villosa *Roth** L., commonly known as Hairy Vetch, is a winter annual weed that belongs to Fabaceae and is native to Europe and Western Asia. Hairy Vetch is used as a cover crop or mulch for weed suppression in organic no-till agriculture due to it allows for an extended window of biomass production in areas restricted to short growing seasons [80,81]. Hairy Vetch was found to have inhibitory effects on the growth of harmful weeds due to its strong ability to compete for light, moisture, nutrients, etc., which is based on the release of the allelochemical, cyanamide [22,82]. Additionally, recent research also found that Hairy Vetch is more tolerant and sensitive to osmotic pressure in plantations than many plant species including *Capsella bursa-pastoris*, *Myriophyllum aquaticum,* and *Avena fatua* [83,84,85,86]. The last among the selected species was *Brassica napus* L., commonly known as Canola and or Rapeseed, which belongs to the Brassicaceae family. Canola is a hybrid species resulting from interspecific breeding between *Brassica rapa* and *Brassica oleracea* [87]. Rapeseed is an economic species and is used as a source of oil and food and as an ornamental plant [88,89]. Although Rapeseed grows well in winter, it is an annual species which depends on growing conditions [90]. Additionally, this bright-yellow flowering is used as a cover crop in the winter season to prevent soil erosion, and as it produces substantial amounts of biomass; it also suppresses weeds and can improve soil tilth with its root system [91]. Calona is not listed as a noxious weed, but their volunteer plants are considered a weed in managed ecosystems which compete with crops for water, nutrients, and sunlight, thus negatively impacting yields [92,93,94].

**Figure 4:**
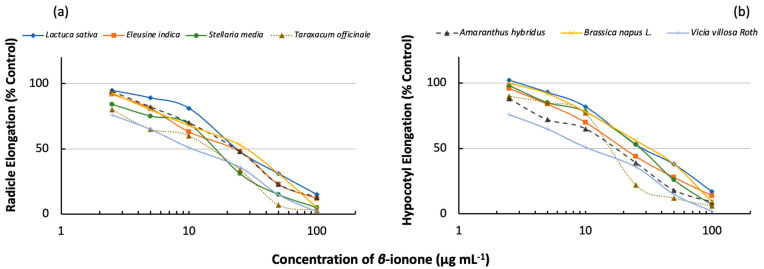
Effect of *β*-ionone on the (**a**) radicle and (**b**) hypocotyl elongation of test plant species. The data are the mean ± standard deviation (SD) of three replications.

Among the test plant species, the content of *β*-ionone significantly inhibited the growths of all plant species as shown in Figure 4, and the inhibition percentage increased with the increasing extract concentrations. The specific activity values (EC_50_) were in the range of 15.4–28.0 µg mL^−1^ and 11.8–31.7 µg mL^−1^ for radicles and hypocotyl elongation, respectively.

The study observed that *β*-ionone possesses phytotoxins strongly enough to suppress the growth of all test plant species when treated with 23 µg mL^−1^. *β*-ionone inhibited the radicle and hypocotyl elongation of *Taraxacum officinale* more than the other test plant species, followed by *Stellaria media*, *Eleusine indica*, *Amaranthus hybridus*, *Lactuca sativa*, *Vicia villosa,* and *Brassica napus*. Recent research showed that *Taraxacum officinale* or Dandelion is an allelopathic plant which had a prominent effect on the germination of *Triticum aestivum* [95]. In this study, the growth of Dandelion was suppressed by *β*-ionone at the concentration of 15.4 and 16.0 µg mL^−1^ for radicle (provide % elongation) and hypocotyl elongation (provide % elongation), respectively. Additionally, this study also observed that *Stellaria media* or Chickweed was suppressed by *β*-ionone at the concentration of 26.4–27.2 µg mL^−1^ and 11.8–31.7 µg mL^−1^ for radicles and hypocotyls elongation, respectively. The Chickweed is known to have an inhibitory effect on wheat growth by the contribution of water-soluble phenolics to the soil [96]. In a different study, *β*-ionone was also found to possess inhibitory effects on the growth of *Microcystis aeruginosa* NIES-843, and the specific activity (EC_50_) was found to be 21.2 ± 1.9 µg mL^−1^. The reaction centre of PS II and electron transport at the acceptor side of PS II are the targets responsible for the toxicity of *β*-ionone based on the transcript expression of genes, polyphasic chlorophyll *a* (Chl *a*) fluorescence transients, and ultrastructural examinations using TEM [97].

Also, this study showed that the radicles were inhibited more than hypocotyl elongations for all the test plant species. These findings were consistent with previous results, which showed low mitotic division at the radicle apex presented in higher radicle inhibition in lettuce when treated with False Yellowhead (*Dittrichia viscose* L.) leaf extracts [98]. Several other studies have also reported similar results using different plant materials [99,100,101,102]. Generally, the radicles are more inhibited than hypocotyl elongation because radicles are the first organs to absorb allelochemicals from the environment [103], and the permeability of allelochemicals into radicle tissue is higher than that of hypocotyl [104]. The results indicated that the *β*-ionone concentration contained in the rhizome of Sweetie Iris has strong allelopathic effects on the growth of other species.

## 3. Conclusions

This study reported the plant growth inhibitory effects of Sweetie Iris crude extract and the corresponding compound *β*-ionone. At the concentration of 23.0 µg mL^−1^, *β*-ionone inhibited 50% of the growth of many weeds and plant species. Hence, *β*-ionone was identified as a bioactive compound in Sweetie Iris, which is responsible for strong allelopathic effects on other species. This study could be used as a piece of baseline information for future evaluation of the effects of *β*-ionone on intact plants and its mode of action, including the application to practical agriculture by mixed planting of Sweetie Iris or exploiting new plant active chemicals as a derivative of *β*-ionone.

## 4. Materials and Methods

### 4.1. Plant Samples and Chemicals for the Bioassay

**Figure 5:**
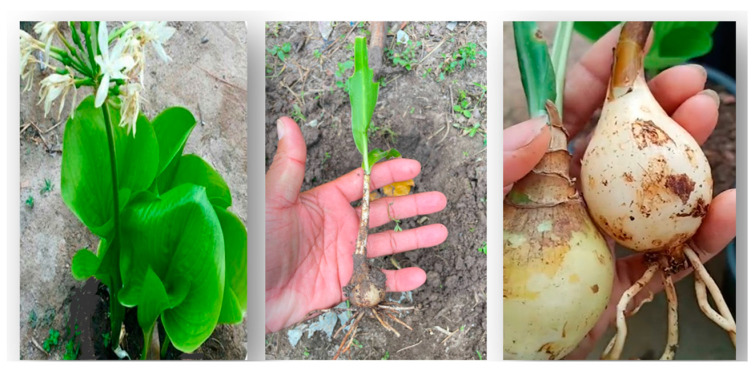
Photo of *Iris pallida* Lam. and its rhizome that was taken from PKNP, Cambodia.

The rhizome of Sweetie Iris (*Iris pallida* Lam.) was collected at the Phnom Kulen National Park (PKNP) (Figure 5), Cambodia which is a known place of medicinal and cultural values in the north-western part of the country. The national park is in the two districts of Banteay Srey and Svay Luer of Siem Reap province. The area is elevated up to 500 m and falls under two main classes, evergreen forest and deciduous forest, which were believed to be the birthplace of the Khmer Empire more than 1200 years ago. The three main aspects that make the national park an important place for conservation are critical ecological attributes, ecosystem services, and social functions. PKNP is approximately home to 1300 to 1500 flora species; however, only 775 species were reported in taxonomy [105]. Recent studies have shown that at least more than 70 plant species were found to have allelopathic potentials; *Iris pallida* demonstrated high inhibitory effects among the 195 screening plant species evaluated using the sandwich and dish park method [39,40].

Sweetie Iris rhizome samples were dried in an oven at 60 °C for 3 h at PKNP before being transported to the Laboratory of the International Agrobiological Resources and Allelopathy, Tokyo University of Agriculture and Technology, Japan, for experimentation. The collected plant materials were authorised by the Ministry of Environment of Cambodia under the national ABS regulation system. All plant materials were exported to Japan through the quarantine system. Lettuce (*Lactuca sativa* L.) was selected as a test plant material in the bioassay due to its reliability in germination and its susceptibility to inhibitory and stimulatory chemicals [106]. The pure compound (*β*-ionone) was purchased from Tokyo Chemical Industry (TCI, Tokyo, Japan). Seven seed plant species were selected and purchased from seed companies in Japan, including TAKII SEED (Kyoto, Japan), SNOW BRAND SEED Co., Ltd. (Hokkaido, Japan), and SAKATA SEED Corp. (Yokohama, Japan). The seeds belong to six representative families, including *Eleusine indica* (L.) Gaertn (Poaceae), *Stellaria media* (L.) Villars (Caryophyllaceae), *Taraxacum officinale* L. and *Lactuca sativa* L. cv. Legacy (Asteraceae), *Amaranthus hybridus* L. (Amaranthaceae), (Apiaceae), *Vicia villosa Roth* L. (Fabaceae), and *Brassica napus* L. (Brassicaceae).

### 4.2. Bioassay for the Phytotoxic Activity of Sweetie Iris and β-Ionone

Oven-dried rhizome of Sweetie Iris (100 mg) was soaked in a glass percolator with 10 mL of MeOH for 48 h at room temperature. The solution was filtered through No.1 filter paper (Advantech Toyo Roshi Kaisha, Tokyo, Japan), centrifuged (13,000 rpm, 10 min), and after that, the supernatants were collected. The specific activity of crude extracts was evaluated using lettuce (*Lactuca sativa* L.) as a test plant material. The original crude extract was diluted to the following concentrations: 0.05, 0.1, 0.25, 0.5, 1, 2, 5, and 10 mg mL^−1^. Filter paper (27 mm ø, Toyo Roshi Kaisha, Ltd., Tokyo, Japan) was placed in a glass Petri dish (27 mm ø). A total of 0.7 mL of the test solution was added to the filter paper and dried completely in vacuo. Five pre-germinated seedlings were placed on the filter paper, and 0.7 mL of distilled water was added and incubated (CN-25C, Mitsubishi Elec., Tokyo, Japan) for three days in dark conditions at a temperature of 22 °C. Three replications were set for each treatment. The control treatment did not have any crude extract but only distilled water. After incubation, the germination percentage of radicle and hypocotyl growth were measured. The phytotoxicity of *β*-ionone on several other selected plant species was also evaluated under laboratory conditions. The inhibitory activity bioassay using *β*-ionone was performed in the same conditions described above, but a total of 0.7 mL of 0.05% Dimethyl sulfoxide (DMSO) test solution was added to the filter paper without drying in vacuo, and the following concentration of crude extracts were 2.5, 5, 10, 25, 50 and 100 μg mL^−1^. Three replications were set for each treatment. The radicle and hypocotyl lengths were measured after the incubation period (the test plant species were measured on day three) and the elongation percentage was calculated using Equation (1), which was modified from Chandra et al. [107].

(1) $$\mathrm{Elongation}\%=\frac{X}{Y}\times 100$$

### 4.3. High-Performance Liquid Chromatography Analysis (HPLC)

A total of 50 mg of the rhizome of Sweetie Iris (*Iris pallida*) was accurately weighed into a 50 mL tube and extracted as shown in the extraction procedure. An aliquot of the extract after centrifugation was filtered through a 0.2 μm syringe filter before injection (10 µL). HPLC analysis was performed using an LC-20AD liquid chromatograph (Shimadzu, Kyoto, Japan). An Itertsil ODS 2 column (250 × 4.6 mm, 5 μm particles, GL Science Inc., Tokyo, Japan) was used. Mobile phases A and B were 0.1% phosphoric with water and MeOH, respectively. The column temperature was maintained at 30 °C, and the flow rate of the mobile phase was set at 0.5 mL min^−1^. The following multi-step gradient with different proportions of mobile phase B was applied: 0 min, 10% B; 5 min, 40% B; 10 min, 80% B; 20 min, 20% B and maintained until 30 min before injection (20 μL). The analysis was monitored using an SPD-M20A detector at 265 nm. Quantification was obtained by comparing the peak areas of the target compounds with the abundance of these compounds in the corresponding standards used in the calibration curve. All chemical analyses were performed in three replications.

## Data Availability

Data are contained within the article.
